# Development of a Sensor with a Lipid/Polymer Membrane Comprising Na^+^ Ionophores to Evaluate the Saltiness Enhancement Effect

**DOI:** 10.3390/s19235251

**Published:** 2019-11-29

**Authors:** Futa Nakatani, Tomofumi Ienaga, Xiao Wu, Yusuke Tahara, Hidekazu Ikezaki, Hiroyuki Sano, Yuki Muto, Yuya Kaneda, Kiyoshi Toko

**Affiliations:** 1 Graduate School of Information Science and Electrical Engineering, Kyushu University, 744 Motooka, Nishi-ku, Fukuoka 819-0395, Japan; ienaga.tomofumi.324@s.kyushu-u.ac.jp (T.I.); y.mutou.666@s.kyushu-u.ac.jp (Y.M.); kaneda.yuuya.735@s.kyushu-u.ac.jp (Y.K.); 2 Research and Development Center for Five-Sense Devices, Kyushu University, 744 Motooka, Nishi-ku, Fukuoka 819-0395, Japan; wu.xiao@nbelab.ed.kyushu-u.ac.jp (X.W.); tahara.yusuke.494@m.kyushu-u.ac.jp (Y.T.); toko@ed.kyushu-u.ac.jp (K.T.); 3 Intelligent Sensor Technology, Inc., 5-1-1 Onna, Atsugi-shi, Kanagawa 243-0032, Japan; ikezaki.hidekazu@insent.co.jp; 4 Fuji Foods Corp., 5-14 Hanedaasahi-cho, Ota-ku, Tokyo 144-0042, Japan; hiroyuki.sano@fuji-foods.co.jp; 5 Institute for Advanced Study, Kyushu University, 744 Motooka, Nishi-ku, Fukuoka 819-0395, Japan

**Keywords:** taste sensor, lipid/polymer membrane, saltiness enhancement effect, saltiness sensor, ionophore

## Abstract

The saltiness enhancement effect is the effect whereby saltiness is enhanced by adding specific substances to salt (sodium chloride). Since this effect can be used in the development of salt-reduced foods, a method to objectively evaluate the saltiness with this effect is required. A taste sensor with lipid/polymer membranes has been used to quantify the taste of food and beverages in recent years. The sensor electrodes of this taste sensor have the feature of selectively responding to each of the five basic tastes, which is realized by the lipid/polymer membranes. In this study, we developed a new saltiness sensor based on the lipid/polymer membrane with the aim of quantifying the saltiness enhancement effect. In addition to the conventional components of a lipid, plasticizer, and polymer supporting reagent, the membrane we developed comprises ionophores, which selectively capture sodium ions. As a result, the response of the sensor increased logarithmically with the activity of NaCl in measured samples, similarly to the taste response of humans. In addition, all of the sensor responses increased upon adding saltiness-enhancing substances, such as citric acid, tartaric acid and branched-chain amino acids (BCAAs), to NaCl samples. These findings suggest that it is possible to quantify the saltiness enhancement effect using a taste sensor with lipid/polymer membranes.

## 1. Introduction

The taste qualities perceived by humans when they taste food or beverages are saltiness, sourness, sweetness, bitterness and umami. These tastes are called the “five basic tastes,” and each taste has a corresponding biological role or feature. The sweetness of, for example, sugar indicates a source of energy for the body, whereas bitterness is a signal of poison. Saltiness, as considered in this study, is the taste conferred by sodium and other ions and indicates the existence of minerals. In addition, saltiness not only contributes to the deliciousness of food but also is routinely used to emphasize the taste of food. On the other hand, salt also has a side effect. As is widely known, an excessive intake of salt is one factor causing lifestyle diseases such as high blood pressure [1,2]. However, if the salt content is reduced, the taste of food becomes bland. Consequently, food with a low salt content but a sufficiently salty taste is desirable. 

The saltiness enhancement effect is the effect whereby saltiness is enhanced by adding specific substances to salt (sodium chloride). Various substances have been reported to be saltiness-enhancing substances contributing to this effect: amino acids such as asparagine and glutamine; branched-chain amino acids (BCAAs) such as valine, leucine and isoleucine, dipeptides such as methionylglycine, sour substances such as tartaric acid and citric acid, protein hydrolysates including amino acids and dipeptides, and so forth [3,4,5,6,7,8,9,10]. In addition, it has been revealed that the flavor of bacon or soy sauce, and spicy substances such as spiranthol, can also enhance the saltiness of food [11,12,13,14,15]. Although there are a wide variety of substances causing this effect, their chemical structures do not have uniformity or commonality, and the mechanism of saltiness enhancement has not yet been clarified. Currently, the development of salt-reduced foods using the saltiness enhancement effect by food industry is in progress. Although the salty taste of salt-reduced food is mainly evaluated by sensory tests, there is no method to quantify the salty taste including the saltiness enhancement effect. In this study, we developed a new taste sensor to evaluate this effect by combining an existing electronic tongue system with an ion-selective electrode.

Sensors that quantify tastes such as electronic tongues (e-tongues) and taste sensors have been developed worldwide, and some of them have already been utilized in food and pharmaceutical industries [16,17,18,19,20,21,22]. The development of e-tongues has almost entirely been based on potentiometric, voltammetric or conductivity-based technologies [23,24,25,26,27,28,29]. E-tongues are suitable for comparing and distinguishing known samples; hence, they greatly contribute to the quality control of food. A taste sensor developed in Japan is called an “electronic tongue with global selectivity” because of this characteristic feature [16,17]. Global selectivity means that this sensor can act as a human gustatory system with selectivity to each taste quality rather than individual chemical substances. The lipid/polymer membrane used in the sensing part of the taste sensor is composed of a lipid, plasticizer and polyvinyl chloride (PVC) as a polymer supporting reagent. The taste sensor outputs a change in membrane potential caused by electrostatic and hydrophobic interactions between the lipid/polymer membrane and the taste substances. Lipids and plasticizers play the roles of adjusting the charge and hydrophobicity of the membrane surface, respectively. In addition to the five basic tastes, this sensor can also quantify astringency, which is perceived via physical stimulation, and synergetic and inhibitory effects of taste [19,30]. Depending on the type and amount of lipids and plasticizers, each sensor electrode responds to one taste quality selectively [18,19,20,31].

A taste sensor has lipid/polymer membrane sensors that can selectively respond to each taste. The sensor for saltiness is named CT0, which is mainly composed of positively charged lipids. The lipids respond to chloride ions when the measurement target is sodium chloride [32]. However, it has been found that CT0 cannot measure the saltiness enhancement effect. To quantify this effect, the detection of sodium ions, which are the source of salinity, was the first issue to be considered. Therefore, bis-crown ether, which is an ionophore used in sodium ion-selective electrodes, was mixed into the lipid/polymer membrane. Ionophores are generally hydrophobic compounds with low molecular weights and function in transmitting specific ions [33]. This bis-crown ether selectively captures metal ions whose sizes match the hole diameter of two crown rings, and forms a sandwich complex. Usually, bis(12-crown-4) is used for sodium ions and bis(benzo-15-crown-5) is used for potassium ions. A sodium ion-selective electrode (NaISE) is composed of bis(12-crown-4) as an ionophore, a plasticizer, an anion exclusion material and PVC, and its sensitivity to sodium ions is 100 times higher than that to potassium ions [34].

In this study, we fabricated a lipid/polymer membrane comprising ionophores by adding bis(12-crown-4) to a membrane based on phosphoric acid di-n-decyl ester (PADE), which is a negatively charged lipid used in other sensor electrodes [19]. The sensor membrane shows the potential response mechanism induced by ionophores and the membrane potential generation mechanism of the lipid/polymer membrane of a conventional taste sensor. Using the fabricated sensor, we attempted to measure the saltiness enhancement effect.

## 2. Experimental Section

### 2.1. Sensory Test

We performed a sensory test on the saltiness enhancement effect in collaboration with the Taste & Aroma Strategic Research Institute Co., Ltd. (Tokyo, Japan). The basic taste samples (salty, sour, umami, bitter) and amino acid samples shown in Table 1 were prepared as base samples. Then, the saltiness intensities of the NaCl sample (saltiness sample in Table 1) and the NaCl + taste samples (sourness, umami, bitterness for medicine, bitterness for food and amino acid samples in Table 1) were compared. Three concentrations of the basic taste samples excluding the saltiness sample were set by taking into account their thresholds, i.e., a third of the threshold, the threshold and three times the threshold [35,36,37,38]. The concentration of amino acids (15 mM) was decided with reference to the following paper [3]. The concentration of NaCl (1%) corresponded to that of typical miso soup in Japan.

Ten well trained panelists (four male and six female adults) were enrolled in this study. The panelists were selected by their ability to detect taste substances with high sensitivity, and have been trained approximately once a week. The sensory test was a two-point identification test in which each panelist selected the sample they felt was saltier. First, the panelists rinsed their mouth with pure water. Then, they were asked to place a sample in their mouths (NaCl + bitterness samples were spat out instead of being swallowed). Two samples were evaluated: first the NaCl sample, then the NaCl + taste sample. Also, when they moved on to the evaluation of the next sample, they washed their mouth with pure water again so that the aftertaste of the previous sample did not remain. Finally, they were asked, “Which sample do you feel was saltier?”. The panelists were given three choices of answer: “NaCl sample”, “Comparable saltiness”, and “NaCl + taste sample”. The compositions of all the samples were not told to the panelists in advance.

### 2.2. Reagents

Bis[(12-crown-4)methyl] 2-dodecyl-2-methylmalonate, 2-nitrophenyl octyl ether (NPOE), tetrakis[3,5-bis(trifluoromethyl)phenyl]borate sodium salt dehydrate (TFPB) and PVC were purchased from FUJIFILM Wako Pure Chemical Corporation (Osaka, Japan). Phosphoric acid di-n-decyl ester (PADE) was purchased from Tokyo Chemical Industry Co., Ltd. (Tokyo, Japan). Sodium chloride (NaCl), L-valine, L-leucine L-isoleucine, L-tartaric acid and citric acid monohydrate were purchased from Kanto Chemical Co., Inc. (Tokyo, Japan). All aqueous solutions used in experiments were prepared with deionized water.

### 2.3. Lipid/Polymer Membrane

We fabricated a sensor with a lipid/polymer membrane comprising ionophores as a new sensor for measuring the saltiness enhancement effect, and a NaISE to compare its response to NaCl with that of the fabricated sensor. The NaISE was composed of bis(12-crown-4) (18 mg) as an ionophore, NPOE (350 µL) as a plasticizer, TFPB (3.5 mg) as an anion exclusion material, which improves the selectivity of ISE to cations, and PVC (180 mg) as a polymer supporting reagent. TFPB itself has a negative charge, and hence, anions in sample solutions become less likely to interact with the ISE membrane. 

As mentioned, the lipid/polymer membranes used in commercial taste sensor electrodes are usually composed of a lipid, a plasticizer and PVC. In addition to the three substances, the newly developed lipid/polymer membrane comprising ionophores contains bis(12-crown-4) (18 mg) and TFPB (3.5 mg). In this membrane, PADE (60 mg), which is negatively charged, NPOE (350 µL) and PVC (180 mg) were used as a lipid, a plasticizer and a polymer supporting reagent, respectively.

First, these materials were dissolved in the solvent tetrahydrofuran (THF) and then stirred. Next, the solution was poured into a petri dish with 45 mm inner diameter, and the membrane was formed by volatilizing the THF. In this study, we measured samples with four sensor electrodes with the membranes formed in one petri dish.

### 2.4. Measurement Procedure of Taste Sensor

**Figure 1:**
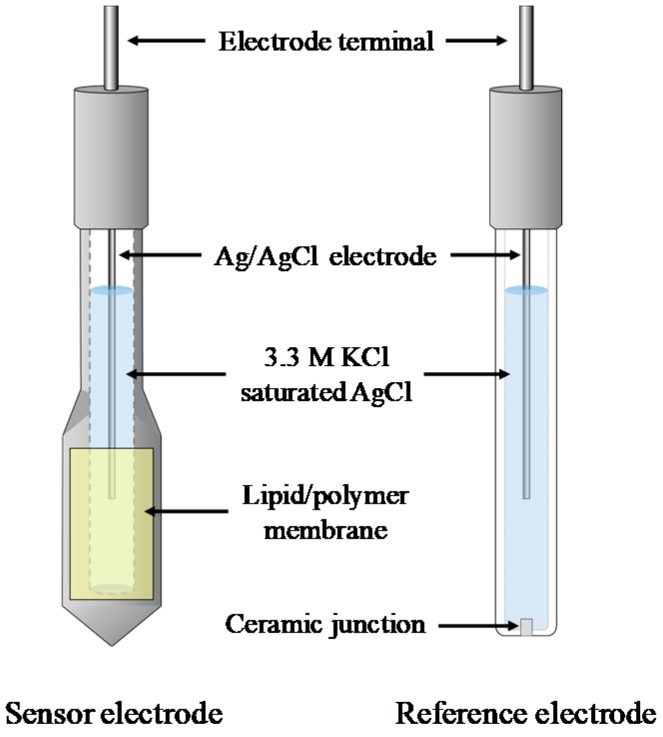
Sensor electrode and reference electrode of the taste sensor.

We used a TS-5000Z commercial taste sensing system (Intelligent Sensor Technology, Inc., Kanagawa, Japan) for measurements. The lipid/polymer membrane comprising ionophores developed in this study or the NaISE was used for sensor electrodes attached to the TS-5000Z. The lipid/polymer membrane was pasted to the receptive parts of hollow bars made of PVC (Figure 1). The potential difference between the sensor electrode and the reference electrode was used as the sensor output.

**Figure 2:**
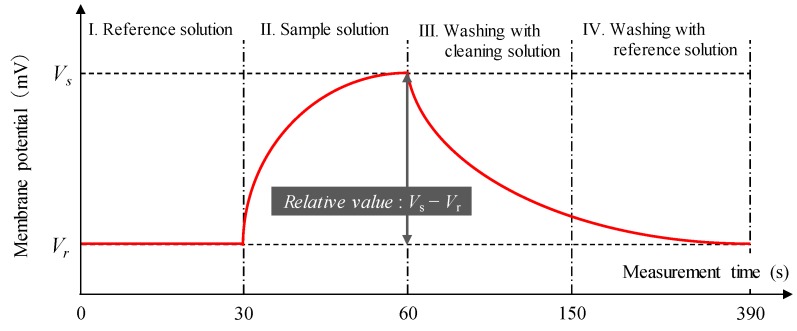
Measurement procedure of taste sensing.

The measurement procedure was as follows [19]. As shown in Figure 2, first, the sensor electrode was soaked in the reference solution (30 mM KCl, 0.3 mM tartaric acid), and the membrane potential (*V*_r_) is measured after 30 s. Next, the sensor electrode was soaked in the sample solution, and the membrane potential (*V*_s_) was measured after 30 s. The difference between *V*_s_ and *V*_r_, (*V*_s_ – *V*_r_), was defined as a relative value. Finally, the membrane surface was initialized by washing with a cleaning solution (100 mM HCl, 30 vol% ethanol) for 90 s and with a reference solution for 240 s. The cleaning solution makes it possible to detach the taste substances adsorbed on to the membrane without leakage of the membrane components and then let the membrane recover to its initial condition. In addition, the membrane potential was confirmed whether it has recovered before Step 1 in Figure 2, and then the next measurement was started. The procedure was repeated five times for each sample using four electrodes, and the average of the second to fifth measurements was calculated as a relative value for all measurements in this study. The standard deviations were calculated for *n* = 4 (electrodes) × 4 (rotations) = 16 values.

### 2.5. Measurement of the Saltiness Enhancement Effect

First, NaCl (0.1–1000 mM) samples were measured using the fabricated sensor with the lipid/polymer membrane comprising ionophores. Then, the responses were compared with that of the NaISE. Next, the saltiness enhancement effects of BCAAs (L-valine, L-leucine and L-isoleucine), citric acid and tartaric acid were measured. BCAAs have been orally administered to patients with cirrhosis or hepatic encephalopathy and as a supplement for fatigue recovery [39,40]. In this measurement, two types of sample, NaCl solution and mixed solutions containing NaCl and a saltiness-enhancing substance, were measured using the sensor with the lipid/polymer membrane comprising ionophores. Moreover, it was investigated whether the sensor response to the mixed solution was larger than that to the NaCl solution. The samples to be measured are shown in Table 2, and the concentration of each saltiness-enhancing substance was prepared on the basis of the result of the sensory test where the saltiness enhancement effect was confirmed. The concentration of NaCl in the sample solutions was set to 100 mM, which is slightly lower than that of miso soup (1%). Deionized water was used as the solvent for all sample solutions.

## 3. Results and Discussion

### 3.1. Sensory Test

**Figure 3:**
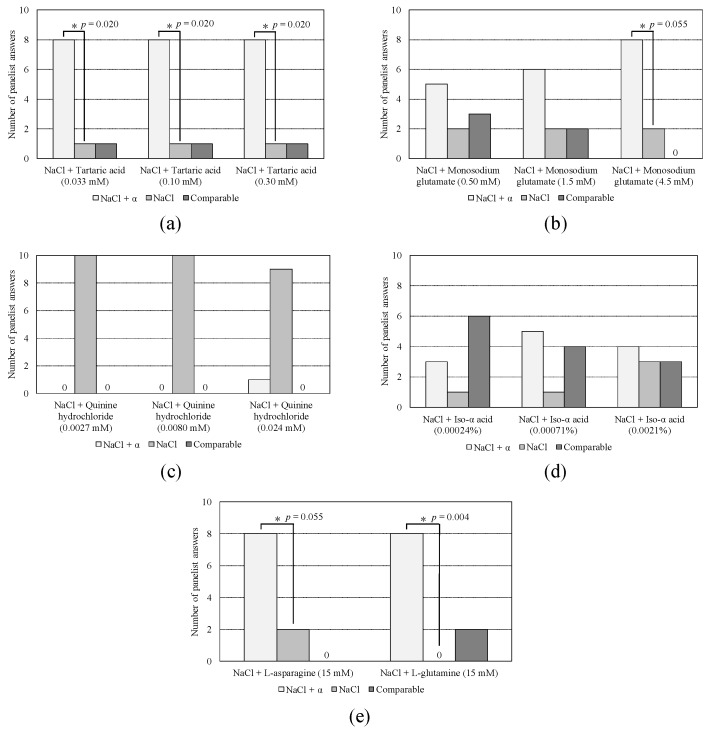
Results of sensory test. α denotes the saltiness-enhancing substance: (**a**) tartaric acid; (**b**) monosodium glutamate; (**c**) quinine hydrochloride dehydrate; (**d**) iso-α acid; (**e**) L-asparagine monohydrate and L-glutamine. *: *p* ≦ 0.05 including *p* = 0.055 (binomial test).

Figure 3 shows the result of the sensory test. The significance of difference was determined by a binomial test using the numbers of panelists who answered “NaCl sample” and “NaCl + taste sample” with *p* ≦ 0.05 indicating a significant difference. In the significance test, although *p* = 0.055 for L-asparagine and monosodium glutamate was larger than 0.05, it was judged to be significant in this test because it was very close to 0.05, and L-asparagine has already been reported to contribute to the saltiness enhancement effect [3]. According to these results, L-asparagine and L-glutamine were shown to contribute to the saltiness enhancement effect as already reported [3]. In addition, tartaric acid and monosodium glutamate contributed to the saltiness enhancement effect.

### 3.2. Response to NaCl

**Figure 4:**
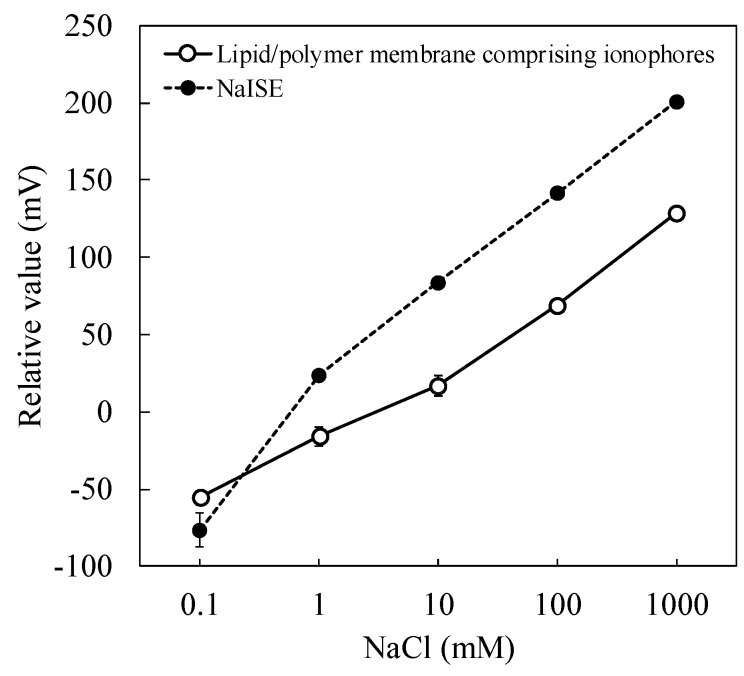
Responses of sensor with the lipid/polymer membrane comprising ionophores and the NaISE to NaCl.

Figure 4 shows the responses of the sensor with the lipid/polymer membrane comprising ionophores and the NaISE to NaCl. The responses of both sensors increased logarithmically with the NaCl concentration. Strictly speaking, the concentration should be replaced by activity. Here we use the word “concentration” for simplicity. The responses of the sensor with the lipid/polymer membrane comprising ionophores and the NaISE increased by 56 mV and 59 mV when the sodium concentration was increased by one order of magnitude in the high-concentration region (10–1000 mM), indicating an ideal Nernst potential response. Although the response of the sensor with the lipid/polymer membrane comprising ionophores was slightly smaller than that of the NaISE, it can be seen that the sensor has sufficient sensitivity to sodium ions. According to the Weber–Fechner law, the intensity of taste perceived by humans increases logarithmically with the concentration of the taste substance. The results shown in Figure 4 satisfy the Weber–Fechner law, and hence the sensor membrane fabricated in this study could quantify the saltiness intensity. Moreover, the use of the sensor membrane for over 100 measurements was possible (CV < 10%).

### 3.3. Response to 100 mM NaCl with Added Saltiness-Enhancing Substances

**Figure 5:**
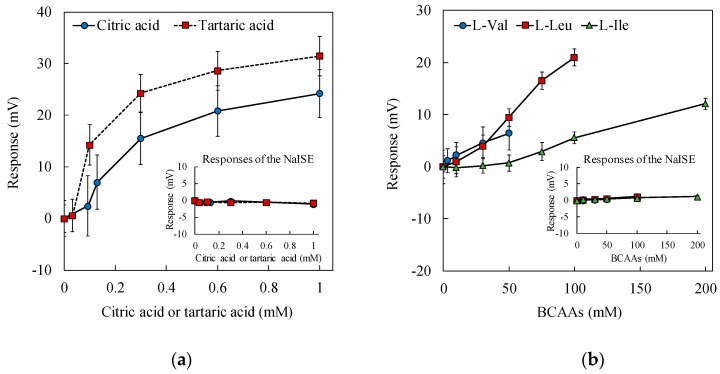
Sensor responses to 100 mM NaCl-based solution with added citric acid and tartaric acid (**a**) and added BCAAs (**b**). The sensor membrane comprises 60 mg PADE.

Citric acid, tartaric acid and BCAAs added to samples measured in this study have all been reported to contribute to the saltiness enhancement effect [4,5,6,8]. For tartaric acid, this was also confirmed in the sensory test of this study. Figure 5a,b shows the responses of the sensor with the lipid/polymer membrane comprising ionophores to each sample. All the samples were based on 100 mM NaCl, and the initial responses of the sensor were standardized to zero (the response to 100 mM NaCl solution was subtracted from all the responses). As shown in Figure 5a,b, the sensor response increased with increasing amount of each saltiness-enhancing substance. On the other hand, the responses of the NaISE to these samples did not increase even when the concentrations of the saltiness-enhancing substances were increased. Since the sensor responses can be considered as an index of saltiness intensity, as shown in Figure 4, the results in Figure 5a,b mean that the saltiness intensity was enhanced with the addition of citric acid, tartaric acid or BCAAs. Thus, it was found that the sensor with the lipid/polymer membrane comprising ionophores can measure the equivalent salt enhancement, and can reproduce and quantify the saltiness enhancement effect.

### 3.4. Response of Sensor with Low PADE Content

**Figure 6:**
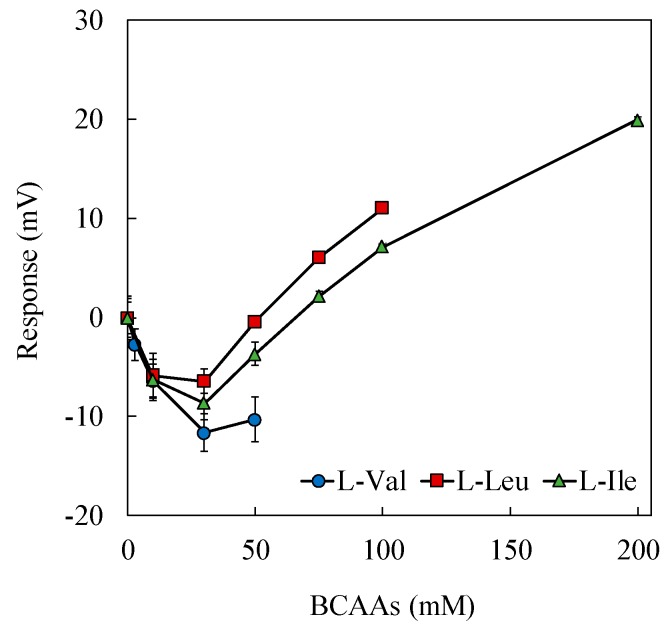
Decrease in sensor response of membrane comprising 40 mg PADE.

As mentioned above, 60 mg PADE, which is a negatively charged lipid, was mixed in the lipid/polymer membrane comprising ionophores in the measurements, to obtain the results in Figure 5. Actually, four sensor membranes comprising 20, 40, 60 and 80 mg PADE (the compositions other than PADE were the same) were fabricated. However, the membrane comprising 80 mg PADE could not be used because of lipid deposition. In addition, the sensor responses of the membranes comprising 20 and 40 mg PADE to the mixed samples of NaCl and BCAAs did not increase monotonically. As shown in Figure 6, the sensor responses of the membrane comprising 40 mg PADE decreased initially, reached a peak at about 30 mM BCAAs, and then increased with the addition of BCAAs.

**Figure 7:**
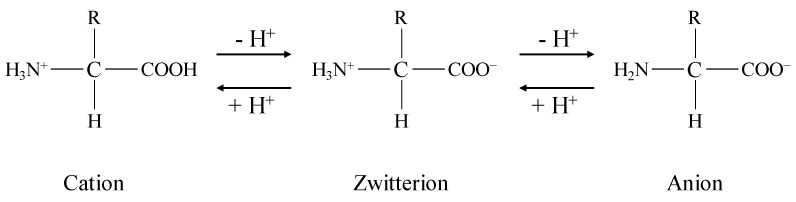
Three ion types of amino acids in solutions.

**Figure 8:**
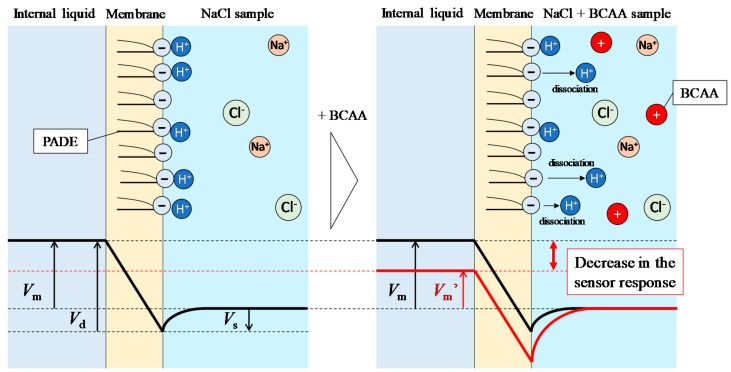
Simple model of decrease in the membrane potential. *V*_m_, *V*_m_’, *V*_s_ and *V*_d_ are the membrane potential, the membrane potential upon adding BCAAs, the surface potential and the diffusion potential, respectively. Ionophores form a diffusion potential inside the membrane owing to sodium ion flow [33]. This figure shows this diffusion potential profile as well as the surface potential originating from charged lipid molecules. Since the diffusion potential is expected to change only slightly, the change in the surface potential is dominant in this situation.

The decreases in the sensor responses are assumed to be caused by the H^+^ dissociation from PADE. The lipid molecule PADE present at the membrane surface is negatively charged by the dissociation of H^+^ in the aqueous solution to be measured. However, not all the molecules of PADE dissociate H^+^; the degree of dissociation depends on the pH and the concentration of cations in the solution [41], and is around 70% in the case of 100 mM NaCl solution [18]. The BCAAs added to the NaCl solution have amino groups and carboxyl groups in their molecules. Amino acids exist in an aqueous solution in the form of the three types of ion shown in Figure 7, where the abundance ratio of each ion depends on the pH of the solution. The pH at which the average charge of an amino acid is zero is called the isoelectric point (Ip). When the pH is lower than the Ip, amino acids receive H^+^, and hence the abundance of cations increases. On the other hand, when the pH is higher than the Ip, amino acids release H^+^, leading to an increase in the abundance of anions. Since the Ip of BCAAs (approx. 6.0) is higher than the pH of NaCl solution (approx. 5.0), BCAAs become cations by receiving H^+^ in the solution. Therefore, the dissociation of PADE is promoted by increasing the concentration of positively charged BCAAs in the measuring sample. As a result, the surface potential of the membrane decreases, and hence the sensor response initially decreases (Figure 8). All the molecules of PADE can be considered to be dissociated at around 30 mM BCAAs, corresponding to the minimum point appearing in Figure 6. Above 30 mM BCAAs, the response potential increased because of the electrostatic interaction between the negatively charged membrane and the positively charged BCAAs. On the other hand, when the membrane comprising 60 mg PADE was used, the sensor response increased with the addition of BCAAs (Figure 5a) instead of decreasing owing to the dissociation of H^+^ from PADE. Therefore, 60 mg of PADE is an appropriate amount to be mixed in the lipid/polymer membrane comprising ionophores for measuring the saltiness enhancement effect.

## 4. Conclusions

With the aim of quantifying the saltiness enhancement effect, we developed a new saltiness sensor, the response of which increases when saltiness-enhancing substances are added to the NaCl sample. The sensor was fabricated with a lipid/polymer membrane comprising ionophores. The sensor response increased logarithmically with the NaCl concentration and this result corresponds to the increasing taste intensity felt by humans. Moreover, the responses to the samples composed of NaCl and a saltiness-enhancing substance (citric acid, tartaric acid or BCAAs) were all larger than the response to only NaCl. We succeeded in developing a new sensor that can reproduce the saltiness enhancement effect. The present work focused on NaCl solutions to which saltiness-enhancing substances were added. Therefore, in future we will measure the effect of other saltiness-enhancing substances or commercial saltiness enhancers. In addition, the application of the sensor to real foods comprising many taste substances and an evaluation of the selectivity, longevity, and repeatability of the sensor are future works, with the ultimate aim of developing a saltiness sensor that can quantify saltiness including the saltiness enhancement effect as an alternative to human sensory tests.

## Figures and Tables

**Table 1:** Compositions of sample solutions to be tested.

Sample	Composition
Saltiness sample	NaCl (1%)
Sourness sample	Tartaric acid (0.033, 0.10, 0.30 mM)
Umami sample	Monosodium glutamate (0.50, 1.5, 4.5 mM)
Bitterness for medicine sample	Quinine hydrochloride dehydrate (0.0027, 0.0080, 0.024 mM)
Bitterness for food sample	Iso-α acid (0.00024, 0.00071, 0.0021%)
Amino acid sample	L-asparagine monohydrate (15 mM) L-glutamine (15 mM)

**Table 2:** Compositions of sample solutions to be measured.

Composition	Concentration
NaCl	100 mM NaCl
NaCl + L-Valine	100 mM NaCl + L-valine (3, 10, 30, 50 mM)
NaCl + L-Leucine	100 mM NaCl + L-leucine (10, 30, 50, 75, 100 mM)
NaCl + L-Isoleucine	100 mM NaCl + L-isoleucine (10, 30, 50, 75, 100, 200 mM)
NaCl + Citric acid	100 mM NaCl + Citric acid (0.094, 0.13, 0.3, 1 mM)
NaCl + Tartaric acid	100 mM NaCl + Tartaric acid (0.033, 0.1, 0.3, 0.61 mM)
